# 

**Published:** 1872-10

**Authors:** 


### CONTENTS.

**COMMUNICATIONS.**

PAGE.

If I were a Doctor. Ben. H. Pratt, A. M. .... 57

Power of Imagination. P. H. Hayes, M. D. .... 59

**CLIPPINGS.**

Illinois Doctor .... 61
Too Little Sleep .... 69
The Food for Infants .... 72
Ingenious Wife .... 72
How Long to Starve .... 82

**CORRESPONDENCE.**

Letters and Answers to Correspondents .... 67

**EDITORIAL.**

PAGE.

Medical Items and News .... 61
Domestic Medicine .... 70
Our Premiums .... 73
Medicine and Doctors .... 73
Magnetic Water .... 74
Strabismus .... 75
Babies, their Food and Sleep .... 77
The use of Tobacco .... 77
Editorial Chit-Chat .... 78
Book Notices .... 83

